# Triboelectric-Enhanced Piezoelectric Nanogenerator with Pressure-Processed Multi-Electrospun Fiber-Based Polymeric Layer for Wearable and Flexible Electronics

**DOI:** 10.3390/polym17172295

**Published:** 2025-08-25

**Authors:** Inkyum Kim, Jonghyeon Yun, Geunchul Kim, Daewon Kim

**Affiliations:** 1 Department of Electronic Engineering, Kyung Hee University, 1732 Deogyeong-daero, Giheung-gu, Yongin 17104, Republic of Korea; 2 Department of Electronics and Information Convergence Engineering, Kyung Hee University, 1732 Deogyeong-daero, Giheung-gu, Yongin 17104, Republic of Korea; 3 Department of Semiconductor Engineering, Kyung Hee University, 1732 Deogyeong-daero, Giheung-gu, Yongin 17104, Republic of Korea

**Keywords:** triboelectric, multi-electrospun fiber, piezoelectric, lateral Janus structure, pressure-treated

## Abstract

A triboelectricity-enhanced piezoelectric nanogenerator (PENG) based on pressure-processed multi-electrospun polymeric layers is herein developed for efficient vibrational energy harvesting. The hybridization of piezoelectric and triboelectric mechanisms through electrospinning has been utilized to enhance electrical output by increasing contact areas and promoting alignment within piezoelectric materials. A multi-layer structure comprising alternating poly (vinylidene fluoride) (PVDF) and poly (hexamethylene adipamide) (PA 6/6) exhibits superior electrical performance. A lateral Janus configuration, providing distinct positive and negative triboelectric polarities, has further optimized device efficiency. This approach introduces a novel operational mechanism, enabling superior performance compared to conventional methods. The fiber-based architecture ensures exceptional flexibility, low weight, and a high surface-to-volume ratio, enabling enhanced energy harvesting. Experimentally, the PENG achieved an open-circuit voltage of 14.59 V, a short-circuit current of 205.7 nA, and a power density of 7.5 mW m^−2^ at a resistance of 30 MΩ with a five-layer structure subjected to post-processing under pressure. A theoretical model has mathematically elucidated the output results. Long-term durability (over 345,600 cycles) has confirmed its robustness. Demonstrations of practical applications include monitoring human joint motion and respiratory activity. These results highlight the potential of the proposed triboelectricity-enhanced PENG for vibrational energy harvesting in flexible and wearable electronic systems.

## 1. Introduction

The rapid proliferation of Internet of Things (IoT) devices has created increasing demands for sustainable energy solutions to address issues related to frequent battery replacements and the environmental concerns associated with discarded batteries [1]. The development of self-powered electronics or the integration of self-charging capabilities into IoT devices is regarded as a promising and eco-friendly alternative [2,3,4]. Among various approaches, ambient energy harvesting, which converts environmental energy from sources such as light, heat, and mechanical motion, has been recognized as a viable strategy [5,6,7].

Among ambient energy sources, mechanical energy, particularly vibrational energy, is considered as a practical and abundant resource due to its widespread occurrence in both natural and artificial environments [8,9]. The principal mechanisms employed for vibrational energy harvesting include triboelectric and piezoelectric effects [10,11,12,13,14,15,16]. Triboelectricity is generated via contact electrification and electrostatic induction when materials with dissimilar electron affinities undergo contact and subsequent separation [17,18]. In contrast, piezoelectricity results from the polarization of piezoelectric materials subjected to mechanical stress [19]. These mechanisms facilitate the conversion of mechanical energy from diverse sources, including wind, ocean waves, rainfall, and biomechanical motions such as respiration, joint movements, and cervical vibrations [20,21].

Electrospinning, a highly adaptable and scalable fabrication method, has been widely employed to create dielectric layers for vibrational energy harvesting devices. Electrospun nanofiber layers offer enhanced surface area, improved alignment of piezoelectric materials, and superior mechanical flexibility, which are critical for practical device applications [22,23,24,25,26,27,28,29]. To further augment the electrical output of electrospun layer-based energy harvesters, a variety of material modifications and structural strategies have been implemented. These include the incorporation of metal-oxide nanoparticles such as ZnO, BaTiO_3_, and PZT [30], as well as carbon nanotubes (CNTs) and graphene [31,32], to reinforce the electrical and mechanical properties of piezoelectric polymers. Structural modifications, such as reducing nanofiber diameters [33], introducing surface microstructures [34], and fabricating porous [35], core–shell [36], and multilayered configurations [37], have also been shown to significantly enhance device performance. Among these approaches, multilayer structures are particularly effective, as they increase both the contact area and fiber density through repetitive fabrication processes.

In this study, a triboelectricity-enhanced piezoelectric nanogenerator (PENG) was developed using multi-electrospun polymeric layers. The proposed device incorporates a triboelectric mechanism within a multilayered structure to achieve superior output performance. Poly (vinylidene fluoride) (PVDF) and poly (hexamethylene adipamide) (PA 6/6) were employed as the active materials due to their contrasting triboelectric polarities, compatibility with electrospinning, and high interfacial adhesion. According to the triboelectric series, tribo-negative polymers such as polyethylene, polyvinyl chloride, polypropylene, and PVDF can be effectively paired with tribo-positive polymers including polyethylene glycol succinate, polymethyl methacrylate, ethyl cellulose, and PA derivatives [38,39]. Among these, PVDF and PA 6/6 offer a favorable combination not only because of their opposite triboelectric tendencies, but also due to their processability and mechanical compatibility in multilayer configurations [40,41,42]. PVDF contributes superior piezoelectric properties [43], whereas PA 6/6 introduces positive triboelectric characteristics. To maximize the synergistic effect of these materials, a lateral Janus structure was designed, featuring distinct triboelectric surfaces. This configuration enhances energy harvesting efficiency during dynamic bending motions and addresses the limitations of conventional single-layer devices.

The operational mechanism of the proposed energy harvesting device has been validated by analyzing charge distributions and conducting finite element simulations. Post-treatment processes, including pressure application with or without heat, were employed to optimize the device’s electrical performance. The triboelectricity-enhanced PENG exhibited significantly improved electrical outputs and durability. The proposed device demonstrated its effectiveness in practical applications, such as monitoring human motion and respiration, thereby confirming its suitability for wearable and flexible electronics [44,45,46,47]. Moreover, the high sensitivity of the device to biomechanical signals suggests potential applications in real-time healthcare monitoring, such as detecting irregular respiratory patterns or tracking daily activity levels. Additionally, the capability to harvest ambient mechanical energy from daily human activities offers a promising step toward reducing dependence on conventional batteries and advancing self-powered wearable healthcare and IoT systems.

## 2. Materials and Methods

### 2.1. Chemicals and Materials

Poly (vinylidene fluoride) (PVDF), poly (hexamethylene adipamide) (PA 6/6), and dimethylacetamide (DMAc) were purchased from Sigma-Aldrich (St. Louis, MO, USA). Formic acid and acetone were procured from Daejung Chemicals & Metals Co. Ltd. (Siheung, Republic of Korea). All chemicals were used as received without further purification.

### 2.2. Synthesis of the Electrospinning Solutions

Electrospinning solutions were prepared by dissolving PVDF and PA 6/6 pellets in appropriate solvents. For the PVDF solution, 0.9 g of PVDF was dissolved in a mixture of 3 mL of DMAc and 2 mL of acetone. The mixture was stirred and heated to 50 °C for 1.5 h to achieve complete dissolution. For the PA 6/6 solution, 0.9 g of PA 6/6 was dissolved in 5 mL of formic acid with continuous stirring at 70 °C for 1.5 h. The solvent compositions and processing conditions were optimized to attain the desired viscosity and fiber uniformity during electrospinning.

### 2.3. Fabrication of the Electrospun Layer

The electrospun layers were fabricated using an electrospinning apparatus from MTDI (Daejeon, Republic of Korea). The solutions were electrospun onto an aluminum substrate under controlled conditions to achieve consistent fiber morphology. For PVDF electrospinning, an input voltage of 10 kV was applied with a solution flow rate of 0.008 mL min^−1^, and the gap between the syringe tip and the substrate was set at 15 cm. For PA 6/6 electrospinning, an input voltage of 19.5 kV and a flow rate of 0.005 mL min^−1^ were employed under the same gap distance. Each layer was deposited for 1 h, and the resulting electrospun samples were left to dry at room temperature overnight to remove residual solvents.

### 2.4. Characterization of Electrospun Samples

The electrospun layers were characterized to confirm their structural and chemical properties. High-resolution field emission scanning electron microscopy (HR FE-SEM, Carl Zeiss MERLIN, Oberkochen, Germany) was performed at 10 kV with a working distance of 6–8 mm to analyze fiber morphology and diameter distributions. Elemental mapping and energy dispersive X-ray spectroscopy (EDS) were conducted to identify the spatial distribution of key elements within the multi-layered structure. FT-IR measurements (ALPHA II, Bruker, Billerica, MA, USA) were carried out in attenuated total reflection mode with a Platinum ATR accessory to analyze the molecular vibrations and bonding states of the layers.

### 2.5. Electrical Output Measurement of the PENG

The electrical output performance of the PENG was measured under controlled vibrational conditions. A gap between the two contact materials was introduced using acrylic foam tape (VHB™, 3M, St Paul, MN, USA). An electrodynamic shaker (LW139.138-40, Labworks Inc., Costa Mesa, CA, USA) was used to apply mechanical forces, with input signals generated by a function generator (33120A, Agilent Technologies Inc., Santa Clara, CA, USA). The shaker frequency and displacement were varied to simulate different operational conditions. Electrical outputs, including open-circuit voltage (*V*_OC_) and short-circuit current (*I*_SC_), were recorded using an electrometer (Model 6514, Keithley Instruments Inc., Cleveland OH, USA) connected to a multi-channel data acquisition (DAQ) system (PCI-6220, NI, Austin, TX, USA).

### 2.6. Finite Element Simulation of the PENG

Finite element simulations were performed using COMSOL Multiphysics 5.0 (Stockholm, Sweden) to visualize the surface electric potential profiles and charge distribution within the PENG. The simulation parameters were calibrated based on the experimental configuration, including material properties and mechanical deformation profiles. These simulations provided insights into the operational mechanism and the synergistic effects of triboelectric and piezoelectric components in the multi-electrospun layers. The transferred charge was obtained by integrating the short-circuit current over time, following baseline subtraction.

## 3. Results and Discussion

### 3.1. Synthesis, Chemical Bonding, and Surface Characterization

**Figure 1:**
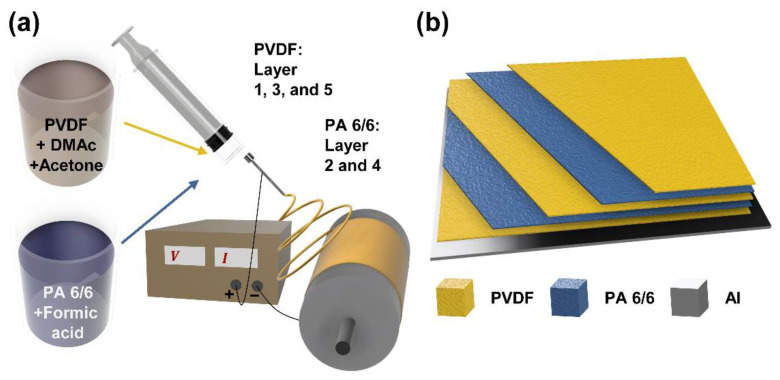
Schematics illustrating the (**a**) electrospinning process and (**b**) resultant multi-electrospun layer.

A schematic illustration of the electrospinning process is provided in Figure 1a, where PVDF and PA 6/6 solutions are sequentially electrospun onto aluminum (Al) foil. Figure 1b depicts the multi-electrospun layer fabricated with a PVDF–PA 6/6–PVDF–PA 6/6–PVDF (F/A/F/A/F) sequence, where yellow and blue indicate PVDF and PA 6/6 layers, respectively.

**Figure 2:**
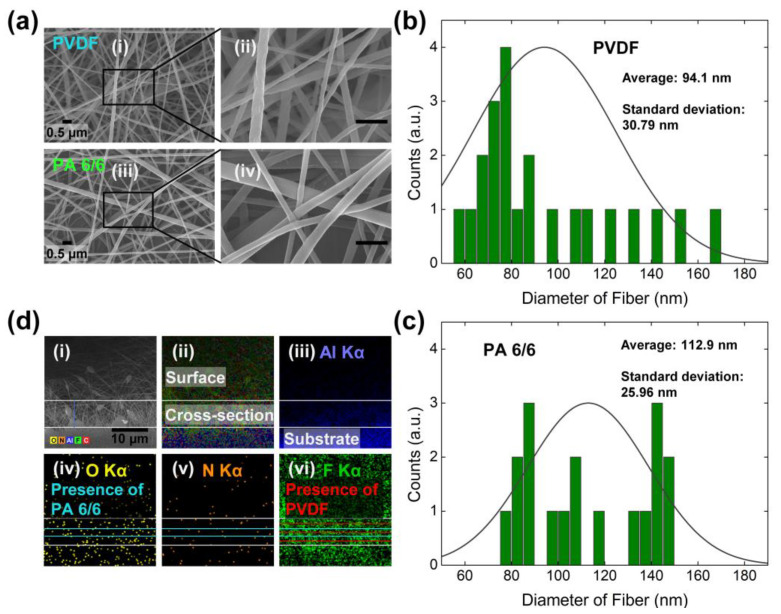
Characterization of the electrospun layer: (**a**) SEM images display the electrospun layer featuring (**i**) PVDF, (**ii**) enlarged PVDF, (**iii**) PA 6/6, and (**iv**) enlarged PA 6/6. Fiber diameter profiles for (**b**) PVDF and (**c**) PA 6/6. (**d**) EDS images detail elemental mapping: (**i**) cross-sectional view of the layer without elemental mapping, (**ii**) with elemental mapping, and the elemental distribution of (**iii**) aluminum, (**iv**) oxygen, (**v**) nitrogen, and (**vi**) fluorine.

Top-view scanning electron microscope (SEM) images of the electrospun PVDF and PA 6/6 layers are shown in Figure 2a. Figure 2a(i) and Figure 2a(ii) display PVDF at magnifications of 30,000× and 100,000×, respectively, while Figure 2a(iii) and Figure 2a(iv) display PA 6/6 at the same magnifications. Both materials form nano- to microscale fibrous networks. The corresponding fiber diameter distributions are presented in Figure 2b,c. The average diameter of PVDF fibers is approximately 94.1 nm with a standard deviation of 30.79 nm, whereas PA 6/6 fibers exhibit an average diameter of 112.9 nm with a standard deviation of 25.96 nm.

Elemental mapping using energy dispersive X-ray spectroscopy (EDS) is presented in Figure 2d. Figure 2d(i) displays a cross-sectional SEM image of the F/A/F/A/F structure, while Figure 2d(ii) shows a composite EDS map that delineates the surface and cross-section of the multi-electrospun layers and the Al substrate using a white boundary line. The individual elemental maps in Figure 2d(iii) to Figure 2d(vi) highlight the distribution of aluminum (Al), oxygen (O), nitrogen (N), and fluorine (F), respectively. The green-marked regions, corresponding to O and N atoms, indicate the presence of PA 6/6, while the blue-marked regions, associated with F atoms, indicate PVDF layers. These results confirm the formation of a five-layered structure. Quantitative analysis from the EDS spectrum (Figure S1) reveals atomic ratios of 23% for F and 1.7% for O within the F/A/F/A/F multilayer.

**Figure 3:**
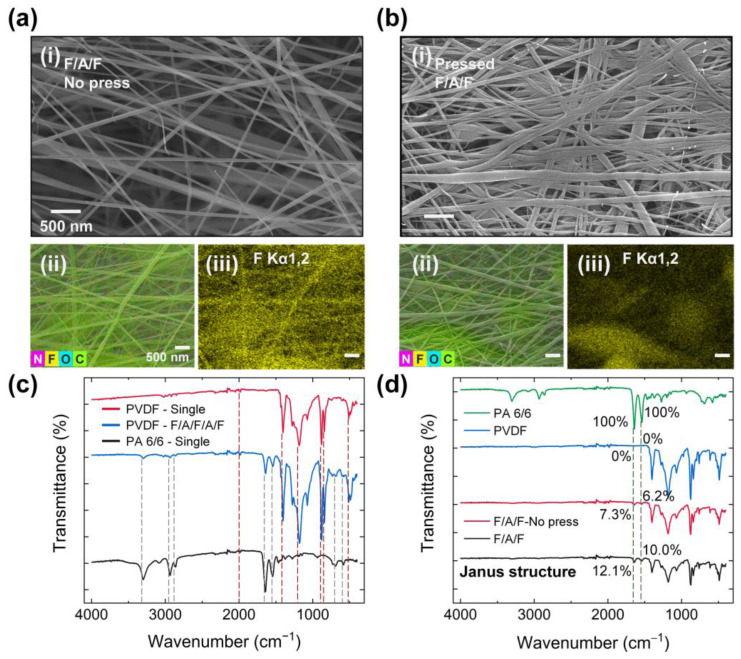
Characterization of the electrospun layer with cold pressing: (**a**) SEM and EDS mapping images display the electrospun layer featuring (**i**) F/A/F layer without pressing, (**ii**) entire element mapping, and (**iii**) fluorine atom mapping. (**b**) SEM and EDS mapping images display the electrospun layer featuring (**i**) F/A/F layer with cold pressing, (**ii**) entire element mapping, and (**iii**) fluorine atom mapping. (**c**) FT-IR spectra comparing the multi-electrospun layer with single material layers. (**d**) FT-IR spectra comparing the multi-electrospun layer with cold pressed layers.

To further enhance the output performance, a cold pressing process was applied to the multi-electrospun layer. SEM and EDS mapping images of the surfaces before and after pressing are shown in Figure 3a and Figure 3b, respectively. After pressing, the nanofiber structure became denser, as observed in the high-magnification SEM and fluorine atom mapping images. A comparison of unpressed and pressed surfaces reveals a 5.88% reduction in the atomic percentage of fluorine and a 3.21% increase in the atomic percentage of nitrogen. This increase in exposed PA 6/6 content is expected to enhance the triboelectric effect, thereby improving the electrical output of the PENG.

Fourier-transform infrared (FT-IR) spectroscopy results are provided in Figure 3c. For the PVDF layer, characteristic peaks at 1182.08 cm^−1^ and 510.11 cm^−1^ correspond to CF_2_ stretching and CF_2_ bending vibrations, respectively [48,49]. X-ray diffraction (XRD) spectra (Figure S2a) confirm the β-phase of PVDF in both single PVDF and unpressed F/A/F configurations [50,51]. For PA 6/6, peaks at 3298.35 cm^−1^, 1640.98 cm^−1^, and 1542.64 cm^−1^ correspond to N–H stretching, C = O stretching, and N–H bending vibrations, respectively [52]. These vibrations are also evident in the F/A/F/A/F structure, confirming the coexistence of both materials, even when PA 6/6 is not exposed at the outermost surface. As shown in Figure 3d, the peak intensities at 1638.93 cm^−1^ and 1536.5 cm^−1^ for PA 6/6 increased by 4.8% and 3.8%, respectively, after pressing. To evaluate piezoelectric response as a function of the PVDF phase composition, XRD analysis was performed on the F/A/F specimens (Figure S2b). The β phase fraction (*F*_β_) was estimated based on Equation (1):

*F*_β_ = *A*_β_/(*A*_α_ + *A*_β_ + *A*_γ_) (1)

where *A*_β_ is the area of the PVDF-β (110/200) reflection integrated over 20.55–20.78°, *A*_α_ is the sum of PVDF-α contributions integrated over phase-PVDF at 20.05–20.35° and 26.1–26.5°, and *A*_γ_ corresponds to the PVDF-γ contribution integrated over 20.05–20.35° [50,51]. The results are summarized in Table S1. The pressed F/A/F samples exhibit *F*_β_ values exceeding 40% (46.8% and 44.6%), markedly higher than that of the unpressed control (27.5%). The increase in *F*_β_ indicates that the simple pressing step promotes formation of the electroactive β phase, consistent with the enhanced piezoelectric response.

Pressing conditions were further optimized by varying both pressure and temperature: 15 MPa (“weak”) or 30 MPa (“strong”) at either 70 °C (hot pressing) or room temperature (RT; cold pressing) for 5 min (Figure S3). Representative top-view SEM images (Figure S3a) and cross-sectional SEM images (Figure S3b) are shown for four pressed samples alongside an unpressed control. The corresponding fiber-diameter distributions extracted from the top-view SEMs are summarized in Figure S3c. On average, pressed samples exhibited ~30 nm larger fiber diameters relative to the unpressed control. Hot-pressed specimens showed slightly larger diameters than cold-pressed ones, consistent with enhanced chain mobility above the glass-transition and phase transition regime of polymer materials under simultaneous heat and load [53,54,55]. Thickness profiles obtained from cross-sectional SEMs (Figure S3d) indicate that pressing reduced the layer thickness to approximately one-half of the unpressed sample. For a given temperature, higher pressure produced thinner layers under cold pressing (from 15 to 30 MPa), whereas thickness under hot pressing became largely insensitive to pressure, suggesting a densification plateau (packing saturation) had been reached at 70 °C over a 5 min dwell time.

To quantify in-plane packing, we computed the fiber area coverage (FAC, %) from five top-view SEM images per condition (Figure S3e). FAC was computed as FAC (%) = 100 × *N*_fiber_/*N*_total_ after percentile contrast stretching (1st–99th) and 3 × 3 box smoothing, followed by global intensity thresholding. A global brightness threshold of 30% (8-bit grayscale) was then applied, yielding a monotonic increase in FAC from 67.2% to 87.4% (Figure S3e, from i to v). These data corroborate the thickness and diameter results: hot pressing promotes tighter packing (smaller inter-fiber voids) than cold pressing, and increased pressure further compacts the mat in the cold-pressed case. Moreover, SEM images of the top-view and cross-section (Figure S3a,b) confirm that the nanofiber structures remained continuous and unbroken after pressing, with no evidence of fiber rupture or inversion of the layered sequence. As no heat was applied during cold pressing, the preserved stack order and mechanical integrity suggest that the layered architecture is maintained. These trends are corroborated by SEM, EDS, FT-IR, and XRD analyses, confirming the structural and chemical modifications induced by the pressing process.

### 3.2. Operating Principle of PENG and Electric Potential Profile with FEM

**Figure 4:**
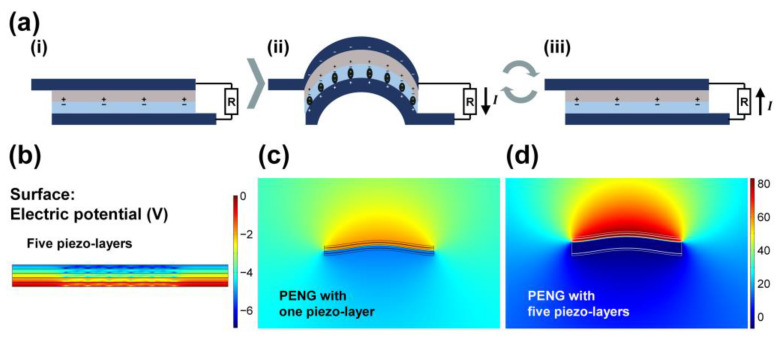
Characterization and performance evaluation of the PENG: (**a**) Operating principle of the triboelectricity-assisted PENG: (**i**) initial, (**ii**) force-applied, and (**iii**) force-released states. Surface electric potential results obtained via FEM: (**b**) five piezoelectric layers, (**c**) PENG with one piezoelectric layer, and (**d**) PENG with five piezoelectric layers.

The operational mechanism of the PENG is illustrated in Figure 4a. In the initial state (Figure 4a(i)), partial contact is established between the top protective layer, connected to the top electrode, and the multi-electrospun layer, affixed to the bottom electrode. Positive and negative charges are generated at the respective contact surfaces. Upon application of external mechanical force, the PENG structure is deformed, leading to curvature in the central region (Figure 4a(ii)). This deformation induces polarization in the piezoelectric material and enhances contact electrification due to the enlarged contact area, resulting in electron flow from the top electrode to the bottom electrode. Upon the release of the applied force, the structure returns to its original flat state, generating a reverse current from the bottom electrode to the top electrode (Figure 4a(iii)). Through this cyclical deformation and recovery process, alternating current (AC) signals corresponding to the input frequency are generated.

To investigate the internal potential distribution and electric field enhancement, finite element method (FEM) simulations were conducted. Figure 4b presents the electric potential profile induced by the polarization of the five piezoelectric layers under an applied force. Compared to the single-layer configuration (Figure S4a), where the electric potential was 2.89 V, the five-layer configuration generated a significantly higher potential of 6.9 V, indicating the synergistic effect of the multilayer structure. Stress and deformation in scenarios with five and one piezoelectric layers are documented in Figure S4b and Figure S4c, respectively.

The potential and surface charge distributions at the electrospun layer of the PENG are further illustrated in Figure 4c,d, corresponding to the configurations with one and five piezoelectric layers, respectively. In the single-layer structure (Figure 4c), a potential of 44.89 V was observed, whereas the five-layer structure (Figure 4d) exhibited an increased potential of 89.82 V. These results demonstrate the effectiveness of the multi-electrospun design in amplifying the internal electric potential and surface charge accumulation, thereby enhancing the overall electrical output of the PENG.

### 3.3. Basic Electrical Output Responses and Material Optimization of PENG

**Figure 5:**
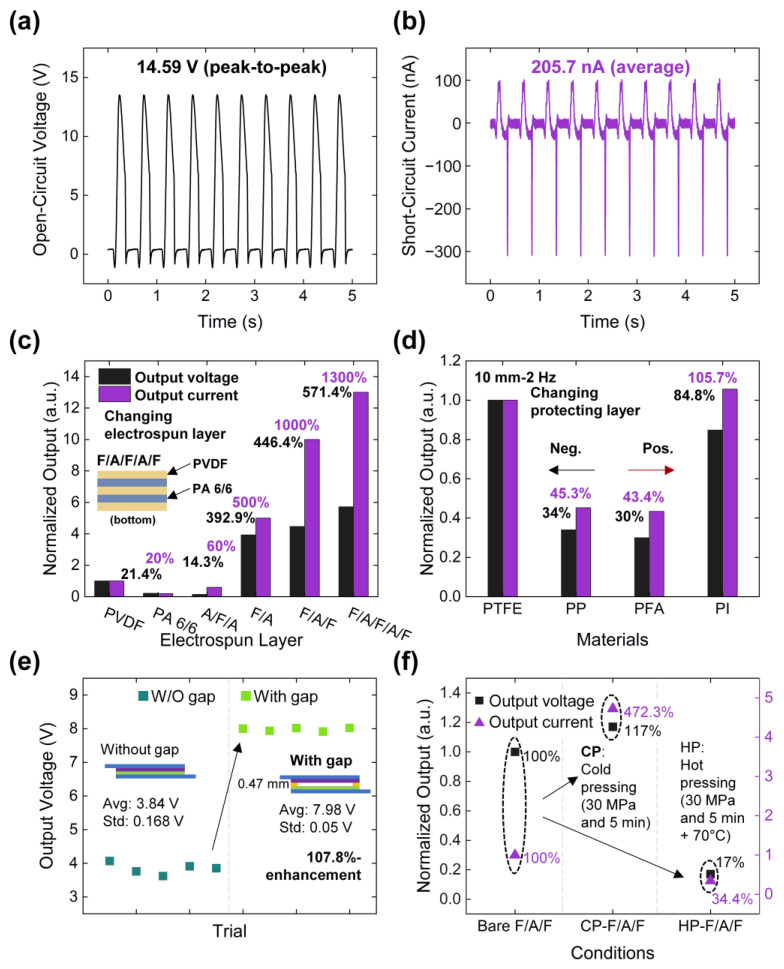
Assessment of the electrical output profiles of PENG: Electrical output signals depicting (**a**) open-circuit voltage and (**b**) short-circuit current. Optimization of materials for improved electrical outputs in (**c**) electrospun layer and (**d**) protecting layer. (**e**) Comparison of output voltage values with and without adding gap between the F/A/F and protecting layers. (**f**) Comparison of electrical output values with and without pressing the F/A/F layer.

The electrical output performance of the PENG was characterized under vibrational conditions at an input frequency of 2 Hz. The measured open-circuit voltage (*V*_OC_) and short-circuit current (*I*_SC_) are presented in Figure 5a and Figure 5b, respectively. The maximum values were recorded as 14.59 V for *V*_OC_ and 205.7 nA for *I*_SC_. The corresponding transferred charge, quantified as 5.01 nC, is shown in Figure S5.

To optimize the device structure, various configurations of electrospun layers were examined, including single layers of PVDF (F) and PA 6/6 (A), and multi-layered arrangements such as A/F/A, F/A, F/A/F, and F/A/F/A/F. As illustrated in Figure 5c, the F configuration exhibited higher electrical output than the A configuration, attributable to the higher dielectric constant and stronger triboelectric charge generation of PVDF. This trend was also observed in multi-layered configurations, with the F/A arrangement demonstrating superior output compared to A/F/A, highlighting the influence of triboelectric polarity distribution. The highest performance was obtained from the F/A/F/A/F configuration. Enhanced performance is attributed to interfacial space-charge (Maxwell–Wagner–Sillars) polarization occurring at PVDF–PA6/6 boundaries, induced by contrasts in dielectric permittivity and electrical conductivity [56]. The accumulated charges intensify the local electric field acting on ferroelectric PVDF domains and, under cyclic loading or poling, promote dipolar alignment. The magnitude of this effect is governed by (i) the degree of permittivity and conductivity contrast and (ii) the total interfacial area, which is determined by the number and thickness of layers.

Similar improvements facilitated by interfacial polarization have been demonstrated in systems beyond PVDF/PA6/6. For instance, in hydroxyethyl cellulose/gelatin (HEC/gelatin) bilayers, a substantial mismatch in relative permittivity (7 and 70 at 1 kHz) induces interfacial charge accumulation, resulting in increased surface charge density and output [57]. In ferroelectric multilayer PVDF-TrFE/BaTiO_3_ nanocomposites, interfacial polarization combined with stress concentration enhances the dielectric constant and current output compared to single-layer structures [58]. These findings suggest a general design principle: property contrast and interface density should be engineered to optimize the performance of electroactive polymer generators.

The proposed model highlights the key features of the system by combining triboelectric, piezoelectric, and interface effects to provide a comprehensive representation of the electrical output voltage (*V_rel_*). This approach accounts for the positional influence of PA 6/6 layers, the dominance of PVDF in enhancing output, and the critical role of interfacial charge distribution. Equation (2) representing this system is:

(2) $$V_{rel}=\sum_{i=1}^{N}\left(\frac{[\alpha \cdot M_{PA,i}\cdot W_{PA,i}+\beta \cdot W_{PVDF,i}](1+\eta g)+d_{33,i}\cdot \sigma_{i}\cdot t_{avg}}{1+k\cdot (N-i+1)}\right)+\lambda \cdot \gamma \cdot N_{Interface}\cdot \Delta Q_{D}$$

where the terms signify distinct physical phenomena. *M_PA_*_,*i*_ reflects the positional sensitivity of PA 6/6 layers: *M_PA_*_,*i*_ = −1 when PA 6/6 is the outermost or single layer and *M_PA_*_,*i*_ = +1 when PA 6/6 is positioned in the middle. *W_PA_*_,*i*_ and *W_PVDF_*_,*i*_ represent the proportional contributions of triboelectric materials, with *d*_33,*i*_·*σ_i_*·*t_avg_* quantifying the piezoelectric polarization using piezoelectric constant, applied stress, and thickness of the electrospun layer. The interface term (*N_Interface_*·Δ*Q_D_*) captures the cumulative interfacial charge dynamics, where *N_Interface_* represents the number of interfaces in the multi-layer structure and Δ*Q_D_* denotes the charge density difference at these interfaces. A linear gap gain factor (1 + *ηg*) is introduced multiplying the triboelectric term, where *g* is the average interlayer gap (in μm) and *η* is the gap gain coefficient (in μm^−1^). In this study, *g* = 0 is set due to measurement limits, and the numerical results remain unchanged; the factor is retained to enable future incorporation of morphology-dependent gains. The coefficients reflect the contributions of individual mechanisms: *α* (triboelectric contribution of PA 6/6), *β* (triboelectric contribution of PVDF), *γ* (interface effect coefficient), *λ* (scaling factor for interface contributions), and *k* (coefficient that controls the saturation rate at sigmoid function). The model is derived under the assumptions of uniform pressure distribution, ideal interfacial contact without slippage, linear elastic deformation, and constant material properties. Edge effects and nonlinearities are neglected for analytical simplicity. Optimization of the model yields *α* = 2.5, *β* = 12.5, *γ* = 0.310, *λ* = 1.004, and *k* = 3, emphasizing the dominant role of PVDF in the output, with secondary contributions from PA 6/6 and interfaces, shown in Figure S6. Using this Equation (2), the relative importance of different mechanisms can be analyzed. The model reveals that PVDF provides 90.37% of the contribution to the output voltage, compared with the 9.63% of PA 6/6.

An electrical short protecting layer was incorporated between the top and bottom electrodes of the PENG to enhance reliability. In Figure 5d, the materials tested for the protecting layer included polytetrafluoroethylene (PTFE), polypropylene (PP), perfluoroalkoxy alkane (PFA), and polyimide (PI). Experimental evaluation of the polarity revealed that PTFE and PP exhibited relatively negative polarity compared to the electrospun PVDF layer at the surface of the F/A/F/A/F configuration. In contrast, PFA and PI displayed reverse polarity. According to the triboelectric series, PTFE and PI layers approximately generated twice the outputs compared to those of PP and PFA, showing the importance of material selection in optimizing triboelectric effects.

To enhance the triboelectric contribution, a 0.47 mm-thick acrylic foam tape was inserted to create a gap between the multi-electrospun layer and the protective layer. As illustrated in Figure 5e, the presence of this gap resulted in a 107.8% increase in output voltage compared to the configuration without the gap. In the absence of the gap, the piezoelectric effect is the predominant mechanism responsible for the electrical output. However, the introduction of the gap facilitates additional electrostatic induction, thereby amplifying the triboelectric effect and synergistically enhancing the overall electrical output performance. The gap distance was further varied to 0.94 mm and 1.88 mm, as shown in Figure S7. Although the output increased with larger gaps, the minimal gap is preferred due to the reduced device volume, which is advantageous for bending and respiration-related applications. Cold and hot pressing techniques were applied to further improve the electrical performance of the PENG. Cold pressing was performed by applying a pressure of 30 MPa to the multi-electrospun layer for 5 min, while hot pressing involved the additional application of heat at 70 °C. In Figure 5f, cold pressing resulted in a 372.3% increase in output voltage and a 17% increase in output current, attributed to the denser nanofiber structure and the exposure of PA 6/6 at the surface, as shown in Figure 3b. However, hot pressing reduced the output voltage and current to 17% and 34.4%, respectively, likely due to PVDF covering the surface of PA 6/6, which diminished the tribo-positive effect of PA 6/6 [59]. This interpretation is supported by SEM analysis (Figure S3), which reveals that hot pressing increased FAC to 87.4%, likely leading to excessive densification and PVDF encapsulation of the PA 6/6 surface. In contrast, cold pressing maintained a more favorable multilayer interface, preserving both piezoelectric and triboelectric contributions. For optimization, the cold pressing method and PTFE protective layer were selected for subsequent experiments, as they yielded the highest electrical performance of the PENG.

### 3.4. Electrical Output Characteristics of PENG with Changing Input Conditions

**Figure 6:**
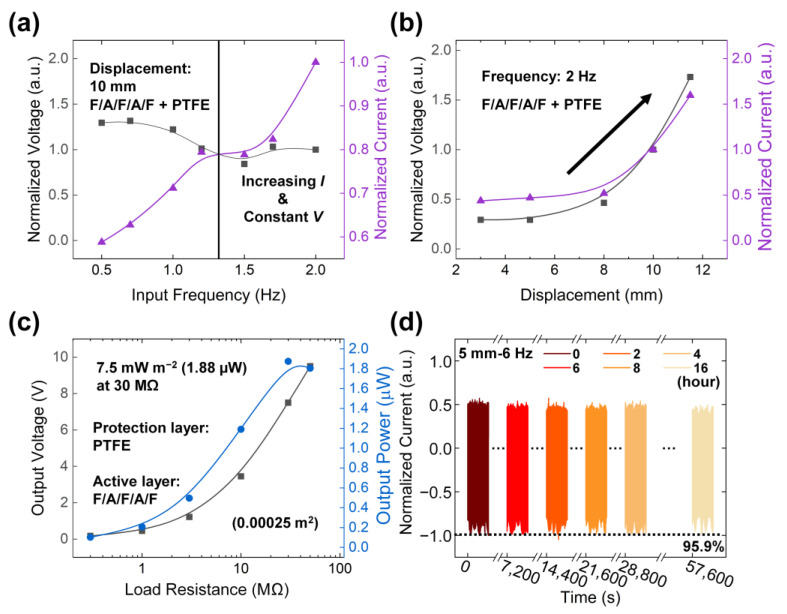
Evaluation and characterization of the PENG: (**a**) Input frequency response and (**b**) intensity response. (**c**) Output power curve of the PENG. (**d**) Durability test results after 345,600 cycles of operation.

The output performance of the PENG was evaluated under varying input frequencies and displacements. As shown in Figure 6a, at a fixed displacement of 10 mm, the output voltage exhibited a slight decrease in the lower frequency region despite maintaining constant input voltage and amplitude from the function generator and amplifier, respectively. While a reduction in output voltage was observed at higher frequencies, the output current increased gradually with increasing input frequency due to enhanced charge transfer rates.

The displacement-dependent response of the PENG at a fixed input frequency of 2 Hz is illustrated in Figure 6b. Both output voltage and current increased proportionally with larger displacements. This enhancement is attributed to the amplified mechanical stress applied to the piezoelectric layers and the intensified contact between the triboelectric interfaces.

The relationship between output power and load resistance was also investigated, as shown in Figure 6c. The maximum output power was determined using Equation (3):

*P* = *V*^2^/*R* (3)

where *P* represents the output power, *V* the output voltage, and *R* the load resistance. As the resistance increased, a corresponding rise in the output voltage was observed, reaching a maximum output power of 1.88 µW, equivalent to a power density of 7.5 mW m^−2^ based on an active area of 1 × 2.5 cm^2^. The optimal load resistance of 30 MΩ corresponds to the internal resistance of the PENG, consistent with the maximum power transfer theorem.

To assess device reliability, a durability test was performed under a continuous input frequency of 6 Hz. As shown in Figure 6d, after 345,600 continuous operation cycles, the output current retained 95.876% of its initial value. The normalized output current was calculated as the average of the absolute values of the positive and negative peak currents. As shown in Figure S8, the PENG exhibited a gradual decline in output voltage and current above 70% RH, generating approximately 80% of its relative performance at 80% RH. Although the output further decreased and became less stable at 90% RH, the device maintained acceptable performance under the 77% RH range, suggesting its potential for use in typical ambient humidity conditions. These results validate the mechanical stability and operational robustness of the PENG under prolonged cyclic loading conditions.

To benchmark the MEL-PENG, it was compared with representative electrospun polymer and hybrid fabric devices that provide similar functionality shown in Table S2 [60,61,62,63,64,65,66]. Compared with prior electrospun PENGs, PVDF–BaTiO_3_ composites typically report ~4.07 mW m^−2^ [60]. BiCl_3_-doped PVDF shows ~2 mW m^−2^ (converted from 0.2 µW cm^−2^) [61]. A dual-doped BiCl_3_/ZnO/PVDF system reaches ~6.4 mW m^−2^ under bending [62]. An aligned-fiber PVDF PENG reports ~2.67 mW m^−2^ [63]. As a triboelectric-only baseline, a cement/GNP TENG delivers about 7 mW m^−2^ under impact excitation [64]; however, this device is not piezoelectric and is included only as a lower-output reference. Collectively, these comparisons indicate that proposed device achieves higher or comparable areal power density while exhibiting markedly superior cycling stability. The enhanced performance is attributed to the lateral Janus architecture, which establishes spatially separated positive and negative triboelectric surfaces. During dynamic bending, this geometry strengthens charge separation and facilitates synergistic tribo–piezo energy transduction, surpassing the capabilities of conventional single-layer designs.

### 3.5. Sensing Demonstration with Human Motion and Respiration

**Figure 7:**
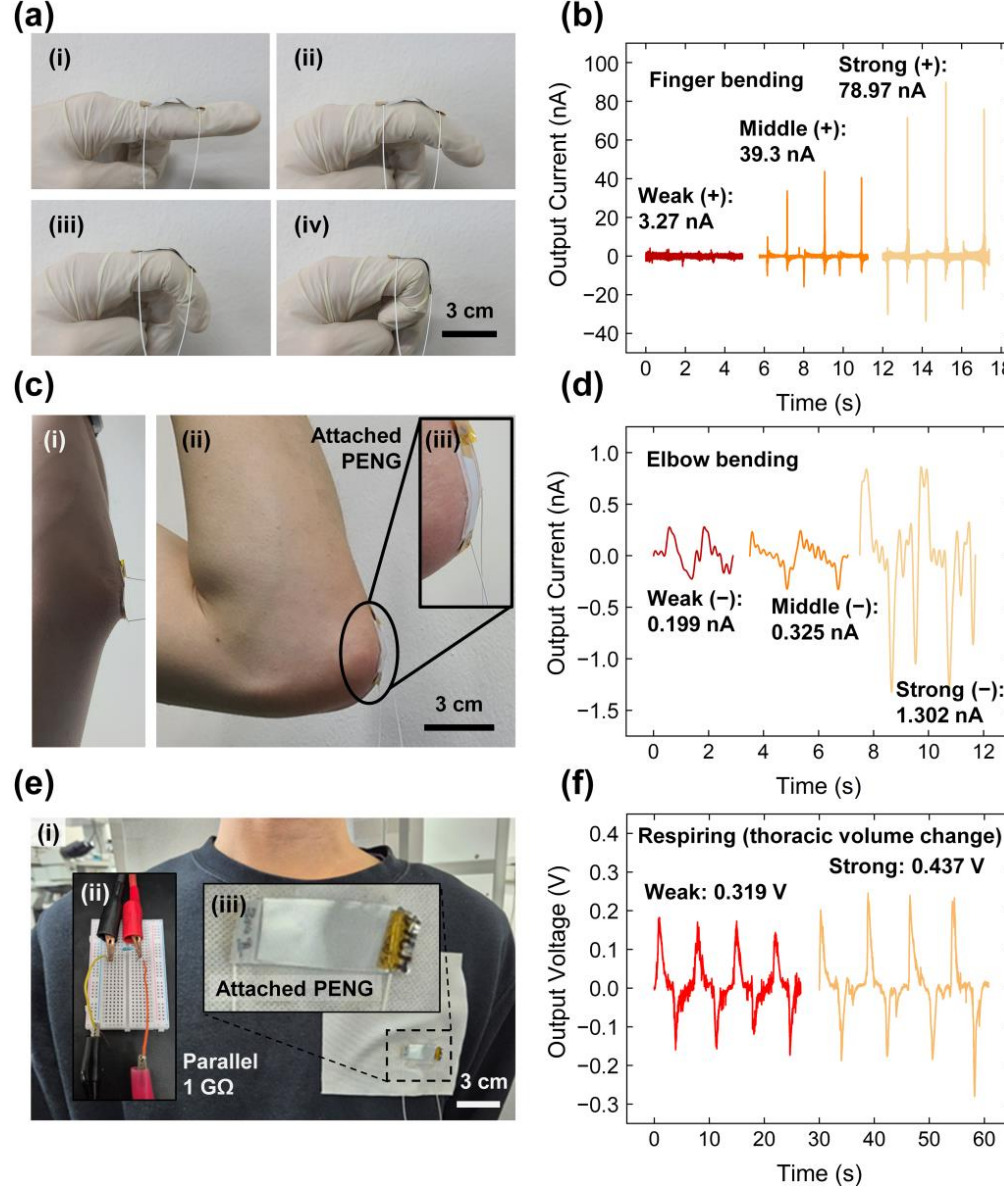
Application of the PENG in detecting bending angles of fingers and elbows: (**a**) Digital camera images of varying finger bending angles: (**i**) 0°, (**ii**) 28°, (**iii**) 58°, and (**iv**) 92°. (**b**) Electrical output signals corresponding to three different finger bending angles. (**c**) Digital camera images of varying elbow bending states: (**i**) flat state and (**ii**) bent state with (**iii**) magnified image of bent state. (**d**) Electrical output signals corresponding to three different elbow bending angles. (**e**) Digital camera images of (**i**) fixed state of PENG at the human chest, (**ii**) connection of the resistance, and (**iii**) magnified PENG. (**f**) Electrical output signals corresponding to two different respiring states.

To demonstrate the practical applicability of the proposed PENG, its ability to detect human motion was evaluated. As shown in Figure 7a, the PENG was attached to a latex glove to function as a flexible sensor for detecting finger bending at angles of 28°, 58°, and 92°. The bending angles were determined from still images by drawing reference lines and computing the included angle. The intermediate angle of 58° represented the fully bent state, while the other angles corresponded to partial and extended bending conditions. The corresponding electrical outputs, as presented in Figure 7b, were measured as 3.27 nA, 39.3 nA, and 78.97 nA for bending angles of 28°, 58°, and 92°, respectively, confirming the device’s high sensitivity to varying mechanical deformations.

The PENG was further applied to monitor larger joint motions by affixing it to the elbow region, as depicted in Figure 7c. Elbow bending at angles of 25°, 90°, and 141° generated output currents of 0.199 nA, 0.325 nA, and 1.302 nA, respectively, as shown in Figure 7d. The relatively lower outputs, compared to finger bending, are attributed to the complex deformation of skin in the elbow area, particularly at the edges, which resulted in less pronounced separation between contact states during flexion. However, the current response curves exhibited a relatively high noise level, which can be attributed to motion artifacts, contact inconsistencies between the device and skin, and possible electromagnetic interference from the environment. To improve the signal-to-noise ratio, the output signal was post-processed using a low-pass filter with a cutoff frequency of 5 Hz, effectively suppressing high-frequency noise components while retaining the main motion-induced signal. Additionally, circuit shielding and more stable mechanical fixation of the device are anticipated to further enhance signal stability in future studies.

To monitor respiratory activity, the PENG was attached to polyethylene (PE) fabric and affixed to the chest area of a shirt, as shown in Figure 7e. The device successfully detected variations in thoracic expansion during respiration. Two distinct breathing conditions—weak and strong respiration—were evaluated, as demonstrated in Videos S1 and S2. As shown in Figure 7f, the output voltages, measured using a 1 GΩ parallel resistance, were 0.319 V and 0.437 V for weak and strong respiration, respectively. These results confirm the capability of the PENG to detect subtle biomechanical signals, validating its potential for wearable respiratory monitoring applications.

## 4. Conclusions

In this study, a triboelectricity-enhanced PENG based on a multi-electrospun layer structure with a protective layer has been developed. The material characteristics of the electrospun layers were thoroughly examined using SEM, EDS, FT-IR, and XRD analyses. SEM results revealed average fiber diameters of 94.1 nm for PVDF and 112.9 nm for PA 6/6. EDS mapping confirmed the presence of fluorine, oxygen, and nitrogen within the multi-electrospun configuration. FT-IR spectra verified the coexistence of characteristic peaks from both PVDF and PA 6/6 in the five-layered F/A/F/A/F structure. The operational mechanism of the triboelectricity-enhanced PENG was validated through FEM simulations and a theoretical model that considered triboelectric, piezoelectric, and interfacial effects. The alternating arrangement of PVDF and PA 6/6 layers, forming a lateral Janus structure, contributed to the generation of distinct positive and negative triboelectric polarities and significantly improved the electrical output. Cold pressing further enhanced device performance by densifying the fiber network and increasing the surface exposure of PA 6/6. The PENG exhibited an open-circuit voltage of 14.59 V and a short-circuit current of 205.7 nA. A power density of 7.5 mW m^−2^ was achieved at a resistance of 30 MΩ. The device maintained 95.876% of its output after 345,600 cycles, confirming its mechanical durability. The PENG demonstrated practical sensing capabilities by detecting joint bending motions and respiratory signals. These results highlight the potential of the proposed device for application in self-powered, wearable, and flexible electronics for energy harvesting and biomechanical monitoring.

## Data Availability

Dataset available on request from the authors.

## Supplementary Materials

The following supporting information can be downloaded at: https://www.mdpi.com/article/10.3390/polym17172295/s1, Figure S1: Energy dispersive X-ray spectrum of F/A/F/A/F layer; Figure S2: X-ray diffraction spectra for PVDF layers; Table S1: PVDF crystallinity analysis; Figure S3: Surface morphology and layer thickness with varying pressing conditions; Figure S4: Finite element analysis of piezoelectric layers; Figure S5: Transferred charge signal from PENG; Figure S6: Fitting of output results and mathematical model; Figure S7: Electrical output with varying gap distance; Figure S8: Humidity response of PENG; Table S2: Comparison of performance and characteristics with previously reported PENGs; Video S1: Video of strong respiration sensing; Video S2: Video of weak respiration sensing.
