# Supplementary material for: Triboelectric-Enhanced Piezoelectric Nanogenerator with Pressure-Processed Multi-Electrospun Fiber-Based Polymeric Layer for Wearable and Flexible Electronics
Source: Polymers (Basel). 2025 Aug 25;17(17):2295. doi: 10.3390/polym17172295 (PMC12431089; doi:10.3390/polym17172295)
Supplement: Supplementary file 1 [file polymers-17-02295-s001.zip › [20250824] (SM) MEL-PENG_polymers-3807862.pdf]

Supplementary Materials

Triboelectric-Enhanced Piezoelectric Nanogenerator with Pressure-Processed Multi-Electrospun Fiber-Based Polymeric Layer for Wearable and Flexible Electronics

Inkyum Kim[1], Jonghyeon Yun[2], Geunchul Kim[3] and Daewon Kim[1,3,*]

[1] Department of Electronic Engineering, Kyung Hee University, 1732 Deogyeong-daero, Giheung-gu, Yongin 17104, Republic of Korea.

[2] Department of Electronics and Information Convergence Engineering, Kyung Hee University, 1732 Deogyeong-daero, Giheung-gu, Yongin 17104, Republic of Korea.

[3] Department of Semiconductor Engineering, Kyung Hee University, 1732 Deogyeong-daero, Giheung-gu, Yongin 17104, Republic of Korea.

* Corresponding author

Email address: daewon@khu.ac.kr (D. Kim)

Table of contents

1. Energy dispersive X-ray spectrum of F/A/F/A/F layer
2. X-ray diffraction spectra for PVDF layers
3. PVDF crystallinity analysis
4. Surface morphology and layer thickness with varying pressing conditions
5. Finite element analysis of piezoelectric layers
6. Transferred charge signal from PENG
7. Fitting of output results and mathematical model
8. Electrical output with varying the gap distance
9. Humidity response of PENG
10. Comparison of performance and characteristics with previously reported PENGs
11. Video of sensing strong respiration
12. Video of sensing weak respiration

1. Energy dispersive X-ray spectrum of F/A/F/A/F layer

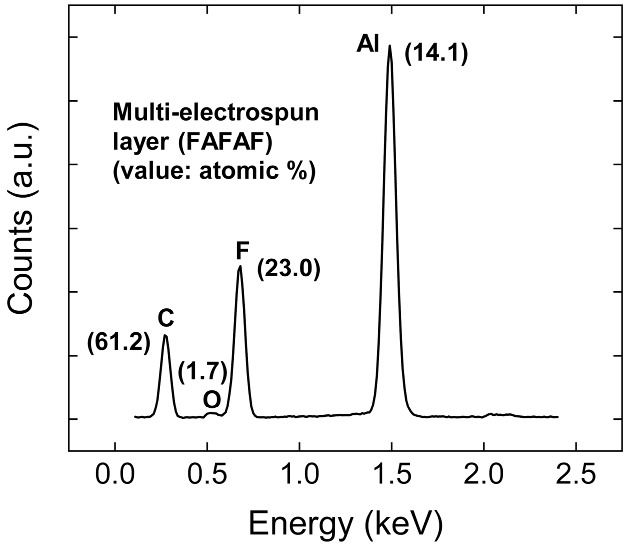

Figure S1. EDS spectrum of the multi-electrospun layer.

2. X-ray diffraction spectra for PVDF layers

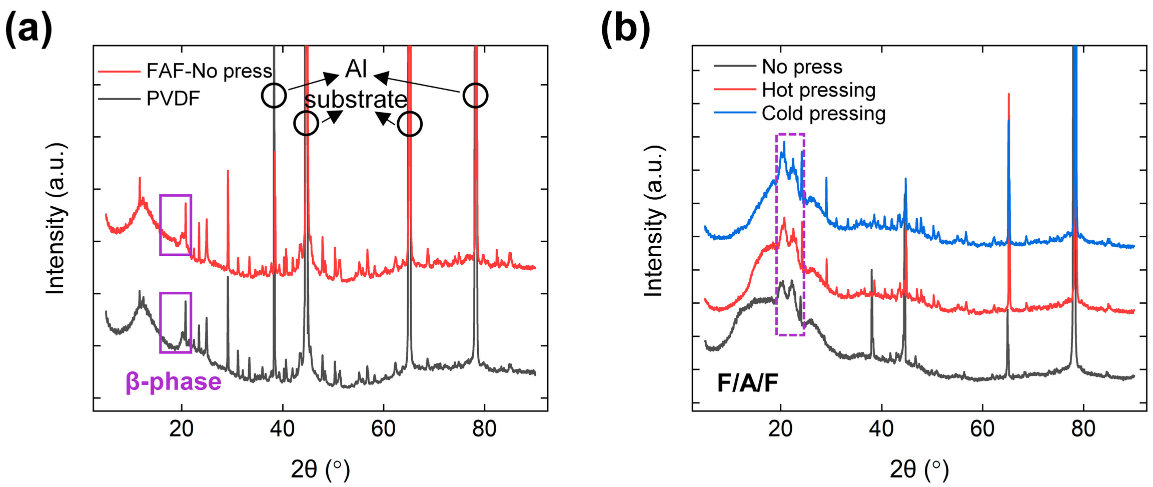

Figure S2. XRD spectra used to evaluate the crystallinity of the surface PVDF layers: (a) multilayer structure and (b) different pressing conditions.

3. PVDF crystallinity analysis

Table S1. Area and fraction values for PVDF in F/A/F samples under different pressing conditions, extracted from XRD spectra.

Sample	A_{β} (20.55–20.78°)	$A_{\alpha/\gamma}$ (20.05–20.35°)	A_{α} (26.1–26.5°)	F_{β} (%)
**Bare**	8.586	16.17	6.427	27.5
**Hot pressing**	23.309	20.294	6.176	46.8
**Cold pressing**	24.834	23.265	7.633	44.6

A: Area under the spectrum, *F*: Phase fraction (Equation: $F_{\beta} = A_{\beta} / (A_{\alpha} + A_{\beta} + A_{\gamma})$).

4. Surface morphology and layer thickness with varying pressing conditions

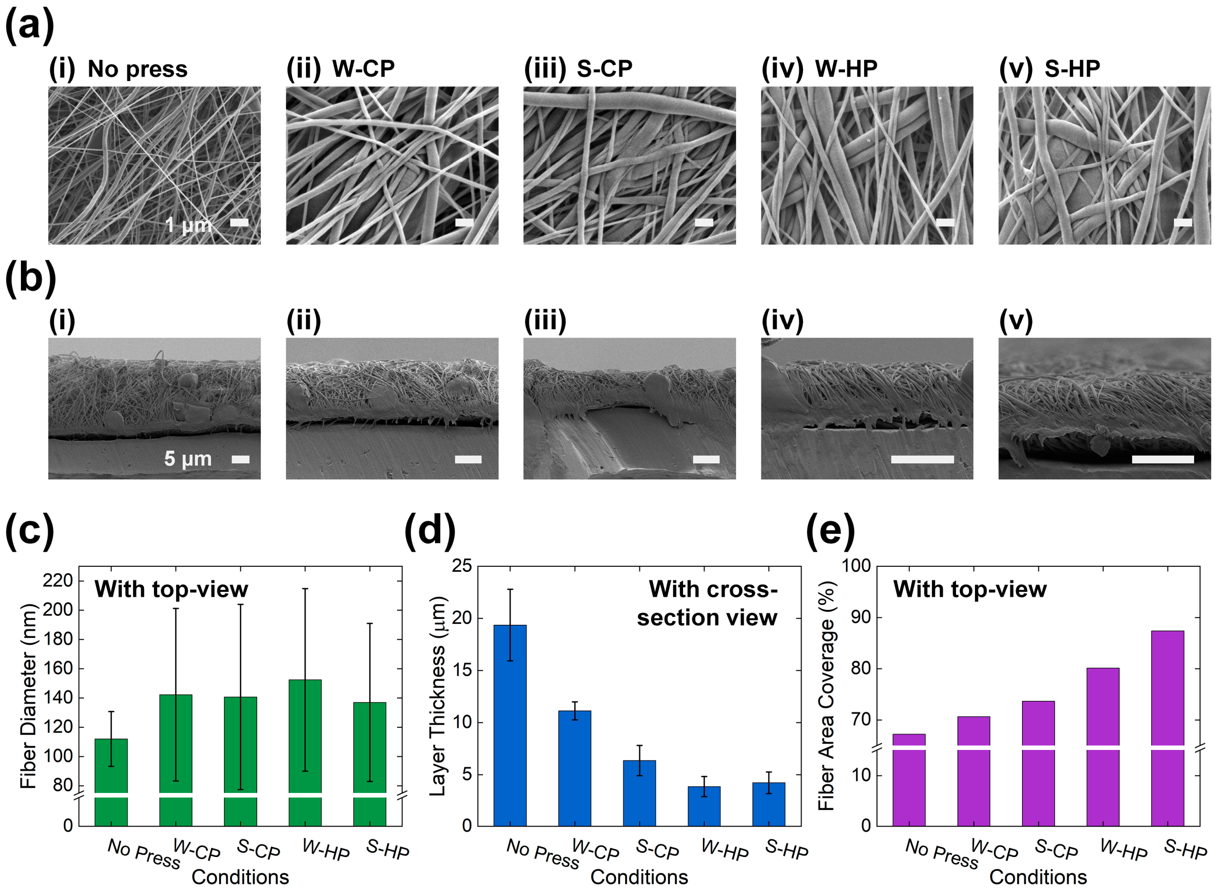

Figure S3. SEM analysis of F/A/F layers under various pressing conditions: (a) top-view images (scale bar: 1 μm), (b) cross-sectional images (scale bar: 5 μm) for (i) no press, (ii) weak pressure (15 MPa)-cold pressing, (iii) strong pressure (30 MPa)-cold pressing, (iv) weak pressure-hot pressing, and (v) strong-pressure-hot pressing conditions. (c) Fiber diameter distribution from top-view SEM images. (d) Layer thickness profile from cross-sectional SEM images. (e) Fiber area coverage quantified via image processing.

5. Finite element analysis of piezoelectric layers

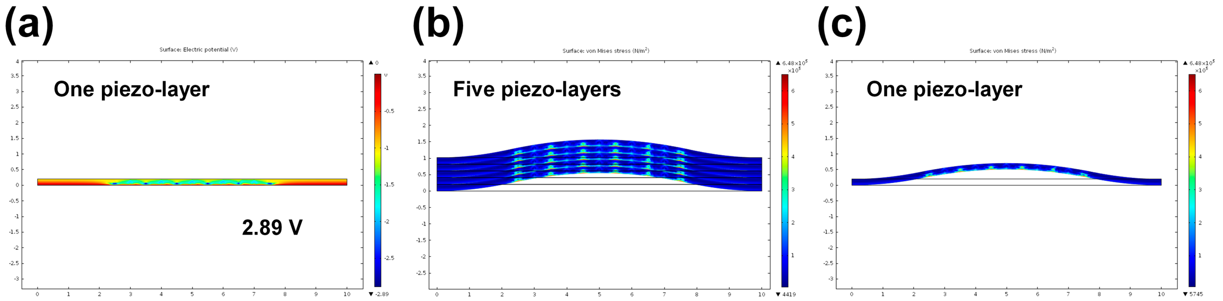

Figure S4. Finite element method (FEM) simulation results: (a) electric potential distribution in a single piezoelectric layer, (b) stress profile across five stacked layers, and (c) stress profile in a single layer.

6. Transferred charge signal from PENG

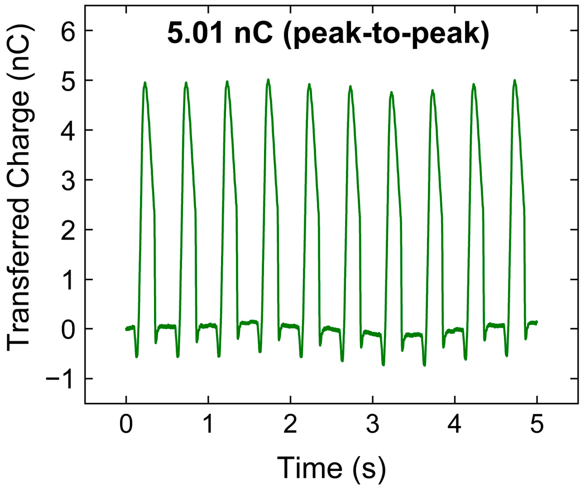

Figure S5. Transferred charge signal generated by the fabricated PENG under an input frequency of 2 Hz.

7. Fitting of output results and mathematical model

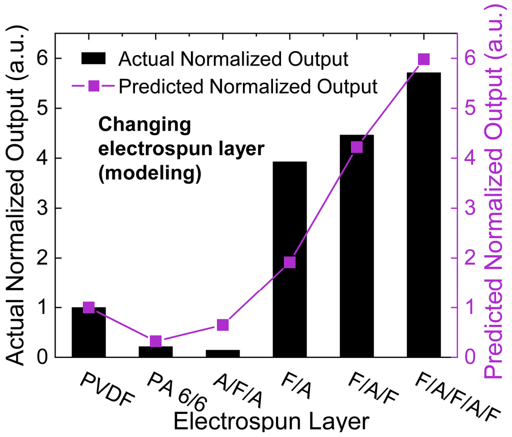

Figure S6. Comparison of relative output voltage between experimental measurements and the predictions from the mathematical model.

8. Electrical output with varying the gap distance

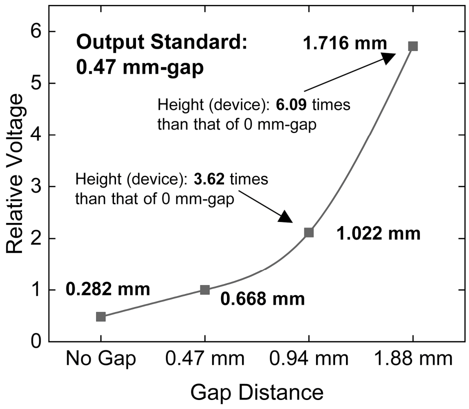

Figure S7. Relative output voltage measured with varying gap distances (0, 0.47, 0.94, and 1.88 mm) between the multi-electrospun layer and the PTFE layer.

9. Humidity response of PENG

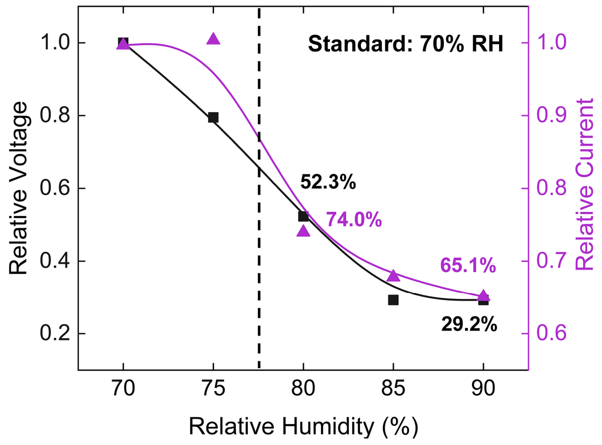

Figure S8. Relative output voltage and current of the MEL-PENG under varying relative humidity conditions ranging from 70 to 90%.

10. Comparison of performance and characteristics with previously reported PENGs

Table S2. Comparative performance metrics of MEL-PENG and representative electrospun or textile-based PENGs reported in the literature.

System (Materials)	Structure / Key Features	Operation Mode	V_{OC} (V)	I_{SC} (μA)	Areal Power Density (mW m^{-2})	Durability	Reference
**This work (F/A/F/A/F, lateral Janus, tribo-assisted PENG)**	Multilayer electrospun; lateral Janus triboelectric surfaces enabling synergistic tribo–piezo harvesting under bending	Bending / vibration	**14.59**	**0.206**	**7.5 @ 30 MΩ**	**95.876% after 345,600 cycles**	This work
**PVDF–$BaTiO_3$ nanofiber PENG (electrospun)**	Electrospun composite (single-mode PENG)	Vibration / bending	~50	0.312 mA m^{-2}	4.07	Stable after ~10,000 cycles	doi:10.1021/acsami.2c07911 [1]
**$BiCl_3$/PVDF nanofiber PENG**	$BiCl_3$ doping to enhance β-phase (single-mode PENG)	Vibration	1.1	2	2	—	doi:10.1016/j.compscitech.2020.108100 [2]
**$BiCl_3$/ZnO/PVDF nanofiber PENG**	Dual dopants; electrospun (single-mode PENG); $d_{33} \approx 3.8$ pC N^{-1}	Bending / vibration	~12	0.34	6.4	—	doi:10.1016/j.eurpolymj.2021.110956 [3]
**Cement/GNP-based TENG**	Triboelectric-only baseline (lower output)	Impact / vibration	330	7	7	—	doi:10.1016/j.nanoen.2025.110823 [4]
**PVDF/Cellulose Acetate-based PENG**	Increased β-phase with cellulose acetate; electrospun	Compression	7.5	2.1	22.6	—	doi:10.3390/ma15197026 [5]
**PVDF/Ce–Fe_2O_3 PENG**	Ce-doped Fe_2O_3 filler; enhanced interfacial polarization	Bending / vibration	20	2.2	—	Stable over 7,200 s	doi:10.1021/acsomega.9b00243 [6]
**Aligned PVDF PENG**	Well-aligned nanofiber structure	Tapping	14	1.22	2.67	—	doi:10.3390/polym13193252 [7]

Performance values are cited directly from the original publications; note that testing conditions and load resistances vary across studies. Areal power density is expressed in mW m^{-2} (1 μW cm^{-2} = 10 mW m^{-2}). A dash ("—") indicates data not reported.

11. Video of sensing strong respiration

Video S1. Measurement of electrical output under strong respiration condition.

12. Video of sensing weak respiration

Video S2. Measurement of electrical output under weak respiration condition.

References

1. Athira, B.S.; George, A.; Vaishna Priya, K.; Hareesh, U.S.; Gowd, E.B.; Surendran, K.P.; Chandran, A. High-Performance Flexible Piezoelectric Nanogenerator Based on Electrospun PVDF-BaTiO3 Nanofibers for Self-Powered Vibration Sensing Applications. *ACS Appl. Mater. Interfaces* **2022**, *14*, 44239–44250, doi:10.1021/acsami.2c07911.
2. Chen, C.; Bai, Z.; Cao, Y.; Dong, M.; Jiang, K.; Zhou, Y.; Tao, Y.; Gu, S.; Xu, J.; Yin, X.; et al. Enhanced Piezoelectric Performance of BiCl3/PVDF Nanofibers-Based Nanogenerators. *Compos. Sci. Technol.* **2020**, *192*, 108100, doi:10.1016/j.compscitech.2020.108100.
3. Zhang, D.; Zhang, X.; Li, X.; Wang, H.; Sang, X.; Zhu, G.; Yeung, Y. Enhanced Piezoelectric Performance of PVDF/BiCl3/ZnO Nanofiber-Based Piezoelectric Nanogenerator. *Eur. Polym. J.* **2022**, *166*, 110956, doi:10.1016/j.eurpolymj.2021.110956.
4. Dong, W.; Duan, Z.; Peng, S.; Chen, Y.; Chu, D.; Tai, H.; Li, W. Triboelectric Nanogenerator-Powering Piezoresistive Cement-Based Sensors for Energy Harvesting and Structural Health Monitoring. *Nano Energy* **2025**, *137*, 110823, doi:10.1016/j.nanoen.2025.110823.
5. Li, Y.; Hu, Q.; Zhang, R.; Ma, W.; Pan, S.; Zhao, Y.; Wang, Q.; Fang, P. Piezoelectric Nanogenerator Based on Electrospinning PVDF/Cellulose Acetate Composite Membranes for Energy Harvesting. *Materials* **2022**, *15*, 7026, doi:10.3390/ma15197026.
6. Parangusan, H.; Ponnamma, D.; AlMaadeed, M.A.A. Toward High Power Generating Piezoelectric Nanofibers: Influence of Particle Size and Surface Electrostatic Interaction of Ce–Fe2O3 and Ce–Co3O4 on PVDF. *ACS Omega* **2019**, *4*, 6312–6323, doi:10.1021/acsomega.9b00243.
7. Jiang, Y.; Deng, Y.; Qi, H. Microstructure Dependence of Output Performance in Flexible PVDF Piezoelectric Nanogenerators. *Polymers* **2021**, *13*, 3252, doi:10.3390/polym13193252.
